# A Feasibility Study of Domain Adaptation for Exercise Intensity Recognition Based on Wearable Sensors

**DOI:** 10.3390/s25113437

**Published:** 2025-05-30

**Authors:** Lei Pang, Yi Li, Ming-Xia Liao, Jia-Gang Qiu, Hui Li, Zhen Wang, Gang Sun

**Affiliations:** 1 Institute of Artificial Intelligence in Sports, Capital University of Physical Education and Sports, Beijing 100091, China; panglei@cupes.edu.cn (L.P.); liyi2021@cupes.edu.cn (Y.L.); l_mingxia@163.com (M.-X.L.); lihui@cupes.edu.cn (H.L.); m17664010653@163.com (Z.W.);; 2 School of Information and Control Engineering, Southwest University of Science and Technology, Mianyang 621010, China

**Keywords:** cross-individual, exercise intensity, ensemble learning, domain adaptation, deep subdomain adaptation network

## Abstract

**Background**: In the fields of rehabilitation, public health, military training and other domains, the accurate and effective monitoring of exercise intensity during exercise can control the occurrence of sports injuries, which is of great significance for people’s healthy lives. **Objective**: This study combined easily collectable multi-dimensional sensor data and various algorithm models to achieve cross-individual recognition of low, middle and high levels of exercise intensity. **Methods**: This study compared the recognition performance of different algorithm models using acceleration and angular velocity sensors worn on seven body parts through individualised body data characteristics. **Results**: The recognition performances of two classical machine learning algorithms were the worst, with a recognition rate of only 82.97% and 80.31%. The performances of two ensemble learning algorithms were slightly better, with a recognition rate of 88.86% and 87.35%. The deep sub-domain adaptation network algorithm proposed in this study exhibited the best performance, with a recognition rate of 92.87%. This study utilised multi-dimensional sensors to construct a cross-individual exercise intensity recognition model for different parts of the body, and the overall recognition rate of the left part was higher than that of the right part. Moreover, the recognition effect upon wearing sensors on the left side of the body is better than the right in running events. **Conclusions**: The results of this study have demonstrated the effectiveness of combining domain adaptation methods and multi-dimensional sensors for cross-individual exercise intensity recognition, laying a solid theoretical foundation for broader-scale cross-individual exercise intensity recognition in future research.

## 1. Introduction

With the rapid development of society, people enjoy greater access to excellent material resources. However, exercise has considerably decreased, leading to various health risks. Reduced exercise has contributed to declining fitness levels and an increased risk of illness [1,2]. Exercise strengthens the body, prevents diseases, reduces obesity, and aids post-injury recovery. It is gradually becoming an indispensable part of daily life [3,4]. However, while exercise brings numerous benefits, it has some drawbacks. For example, improper exercise intensity may lead to decreased immunity, exercise addiction and damage to the body [5,6]. Meanwhile, during the postoperative rehabilitation process, exercise, as a crucial therapeutic approach, requires strict control over its intensity. This is because both insufficient and excessive intensity can cause secondary injuries [7].

Currently, there are many commonly used methods for recognising exercise intensity, such as the rating of perceived exertion (RPE) scale, the talk test (TT), heart rate, motion sensors and oxygen uptake [8,9]. Chai G et al. studied the relationship between the RPE scale and exercise intensity and found that the percentage of heart rate reserve (%HRR) is significantly correlated with RPE. Additionally, %HRR can effectively identify exercise intensity [10]. Porcari J P et al. used the TT method to monitor exercise intensity and found that this method is similar to the %HRR method. They pointed out that TT is a simple and low-cost method [11]. Cowan R et al. studied the effectiveness of TT and RPE in monitoring exercise intensity and found that the measured values of RPE at low and middle exercise intensity were greater than the actual exercise intensity values. In high-intensity exercise, the measurement results were comparable to the actual exercise intensity. Researchers believe that TT is only suitable for measurements during non-strenuous exercise [12]. However, these two methods are difficult to use for high real-time requirements.

Compared to the above-mentioned methods, using wearable devices for exercise intensity monitoring is more feasible. Ho W T et al. used wristband devices to measure the heart rate and estimate exercise intensity. They found that when exercise intensity is low, the wristband devices could accurately reflect the magnitude of exercise intensity. However, as exercise intensity increased continuously, the error rate also increased [13]. Tylcz J B et al. collected acceleration data through a smartwatch to verify the effect of distinguishing exercise intensity and found that this method could differentiate between low, middle and high exercise intensity [14]. Mukaino et al. used lumbar spine accelerometers and heart rate sensors to monitor the activity intensity of paraplegic persons and found a significant correlation between acceleration and heart rate data and activity intensity [15]. In the aforementioned studies, the motion sensors contained abundant movement information, and in some studies, a strong correlation was found between heart rate and exercise intensity. This study will integrate basic information (height, weight, age, gender and resting heart rate) and motion information (triaxial acceleration, triaxial angular velocity and exercise heart rate) as features for identifying exercise intensity.

Artificial intelligence algorithms have shown great advantages in building high-dimensional exercise intensity recognition models based on basic information and motion information. Bai C et al. used machine learning algorithms based on wrist strap acceleration sensors to identify physical activity intensity. They found that machine learning algorithms could effectively recognise the levels of physical activity intensity, with the highest F1-score reaching 0.946, and could estimate the amount of physical activity of the elderly [16]. Garcia-Garcia F et al. used accelerometer and heart rate data to construct linear discriminant analysis and k-means clustering models, and these models afforded good recognition results [17]. Therefore, developing algorithmic models for exercise intensity recognition is considered an effective method. Owing to individual variations, it is challenging to apply models built from the data of a certain group of people to other groups. Moreover, collecting exercise data from all individuals appears to be a difficult task. In domain adaptation techniques, feature distribution alignment methods explicitly align the feature distributions between the source and target domains, effectively addressing the model generalisation issues caused by cross-domain data distribution differences. Cai Z et al. employed electroencephalography for inter-disciplinary and temporal emotion recognition, achieving an accuracy of 58.23% with their Support Vector Machine. The use of maximum classifier difference in domain adversarial neural networks increased the accuracy to 88.33% [18]. Huang D et al. constructed a cross-individual emotion recognition algorithm model through an electroencephalogram, mainly guided by the idea of a generative adversarial network, and performed feature alignment, providing a new idea for cross-individual emotion recognition. This demonstrates that domain adaptation methods hold significant potential in addressing cross-individual issues [19].

**Figure 1:**
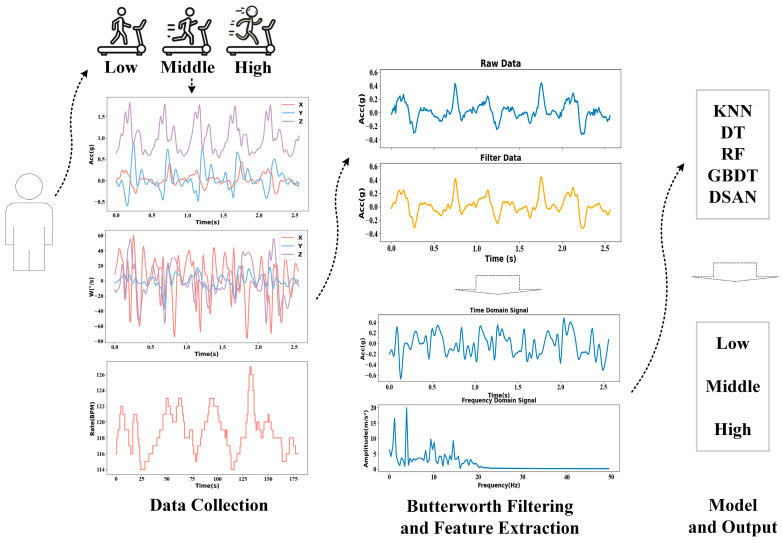
Framework diagram.

The American College of Sports Medicine (ACSM) categorises exercise intensity into five levels based on %VO_2max_. Exercise intensities below 37% are classified as low intensity, 37–45% as light intensity, 46–63% as middle intensity, 64–90% as vigorous intensity and ≥91% as very vigorous to maximal intensity [20]. In daily life, people can achieve low exercise intensity through regular activities, but it is difficult to reach very vigorous to maximal intensity. The ‘Guidelines on Physical Activity and Sedentary Behavior’ issued by the World Health Organization and the ‘Chinese Physical Activity Guidelines (2021)’ issued by the Disease Prevention and Control Bureau of the National Health Commission of China propose that people should perform a certain duration of middle- and high-intensity exercise every week or every day [1,21]. Therefore, this study utilised multi-dimensional data combined with artificial intelligence algorithms to perform cross-individual recognition for low, middle and vigorous exercise intensities, subsequently re-classifying them into the low, middle and high exercise levels. The main work of this study is as follows: (1) collecting multi-dimensional motion data, (2) data cleaning and processing, (3) extracting time-domain and frequency-domain features of three-axis acceleration and three-axis angular velocity data and constructing a dataset and (4) comparing the recognition rates of the classical machine learning algorithm, ensemble learning algorithm and domain adaptation algorithm in different parts of low-, middle- and high-intensity exercises across individuals. The framework of this study is shown in Figure 1.

## 2. Methodology

### 2.1. Participants

In this study, 24 students from the Capital University of Physical Education and Sports (China) participated in the experiment (Table 1). The age span of the participants was small (20–30), and there were significant differences in weight and resting heart rate among women, while the corresponding differences between men were relatively small. The study protocol was approved by the Ethics Committee at the Capital University of Physical Education and Sports. Participants with healthy limbs were recruited, the participants in the experiment had no medical conditions for which exercise was not advised by doctors and they had no open or closed injuries within the past three months. Before the beginning of the experiment, the participants were provided with a detailed introduction to the overall process of the experiment and an informed consent form was signed.

### 2.2. Experimental Equipment

Basic information: In this study, multiple sensor devices were used to collect data, a body measuring ruler was used to measure participant height and a body composition analyser named InBody 270 Analyzer (InBody Co., Ltd., Seoul, Republic of Korea) was used to measure participant weight. OMEGwave (Beijing Yanding Huachuang Sports Development Co., Ltd., Beijing, China) was used to collect the human resting heart rate.

Motion information: The speed of the h/p/cosmos para treadmill (h/p/cosmos sports & medical GmbH, Nussdorf-Traunstein, Germany) was controlled through the serial port to control the power output of the participant. The maximal oxygen uptakes and real-time oxygen uptakes of the participant were measured by the CARDIOVIT AT-104 ErgoSpiro (Schiller International AG; Alfred Schiller (Beijing, China) Medical Technology Co., Ltd., Beijing, China). A Polar H10 heart rate band (Polar Electro Oy, Kempele, Finland; Alfred Schiller (Beijing, China) Medical Technology Co., Ltd., Beijing, China) collected the heart rates of the participants in real time and uploaded them to the APP via Bluetooth, with a collection frequency of 1 Hz. WitMotion BWT901BCL0.5 (WitMotion Shenzhen Co., Ltd., Shenzhen, China) collected triaxial acceleration, triaxial angular velocity and triaxial Euler angle information in real time in the adjustable data acquisition frequency range of 1–1000 Hz.

### 2.3. Experimental Design

The participants did not engage in any strenuous exercise within 24 h before the experiment, and the experiment was conducted at least 1 h after meals. The indoor environment was well ventilated, quiet and tidy. The room temperature was maintained at 20–30 °C, and there were no safety hazards present. To reduce the error caused by the inconsistent orientation of the WitMotion sensor, the WitMotion switch direction was uniformly upward. Previous work showed that the frequency of acceleration and angular velocity should be set to at least 100 Hz [22] during external load monitoring, so we set the WitMotion acquisition frequency to 100 Hz. As the acquisition frequencies of the inertial sensor and HR were 100 and 1 Hz, respectively, and heart rate changes little within a second, the HR data were upsampled to 100 Hz using the zero-order hold method. This involved duplicating each HR value collected per second 100 times to match the data sampling frequency of the inertial sensor. Motion data were collected from three stages of running: low, middle and high intensity. The detailed information is shown in Table 2.

Before the experiment, the experiment procedure and precautions were explained to the participants. After starting the experiment, the basic information of the participants (height, weight, age and gender) was first collected, and OMEGwave was used to measure their resting heart rate. The participants were then fitted with experimental devices (energy respiratory metaboliser and Polar heart rate band) and WitMotion sensors on seven parts of the body, uniformly keeping the sign of the data transmission interface up. The experiment was divided into two, with a time interval of 24 h. The maximal oxygen uptake was measured in the first experiment, and the exercise data for low, middle and high intensity were collected in the second experiment.

First, the VO_2max_ of each participant was measured. After the formal start of the experiment, the participants were instructed to sit still for 5 min. After the resting state was completed, they began to warm up on the h/p/cosmos para treadmill to prepare for the subsequent maximum-oxygen-uptake test. The initial speed of the participants on the treadmill was 7 km/h, and the 1 min increasing rate (1 km/h) was maintained to enter the stage of linear increasing load exercise. The experimenter gave appropriate verbal encouragement according to the physiological performance of the participants during exercise. When any three of the following criteria were met or the participants were subjectively unable to continue the exercise load, the exercise load was stopped immediately: achieving the maximum heart rate (208 − 0.7 × age according to the ACSM’s guidelines for healthy individuals), respiratory entropy (as measured by the instrument) > 1.15, self-perceived exhaustion of the participant and reaching the point of oxygen consumption plateau. Even after reaching exercise exhaustion, the participants continued to steadily jog at 7 km/h for an additional 3 min until the conclusion of the experiment. The maximal oxygen consumption metrics were recorded, and the respiratory mask was disinfected. Following the first experiment, participants were instructed to maintain their regular schedule before the second experiment, such as avoiding staying up late, drinking alcohol, or excessive exercise, to minimise any potential impact on the results.

Subsequently, data for low, middle and high exercise intensities were collected. After the experiment commenced, participants similarly engaged in a 5 min seated rest period, followed by sequential data collection corresponding to the states of low-, middle- and high-intensity exercise. Taking low-intensity data collection as an example, participants gradually increased the load on the h/p/cosmos para treadmill from the lowest speed until their oxygen uptake reached 0–45% of their maximum oxygen uptake and stabilised. They maintained this speed for 4 min and then rested until their heart rate dropped below 80 bpm. For middle-intensity (46–63%) and high-intensity (64–100%) exercises, the above-mentioned protocol was repeated. After the experiment, the exercise data of the participants were recorded, and the respiratory mask was disinfected.

### 2.4. Data Pre-Processing and Feature Extraction

Due to the high sensitivity of the inertial sensors, some high-frequency noise may be generated during data collection, along with artifacts from clothing and similar issues. This study used Butterworth filters to filter the data and set the cut-off frequency to 25 Hz [23]. The duration of collecting exercise data for different intensity stages was 4 min each. Therefore, to improve the accuracy of the data, the data from the first 2/3 min were deleted and then filtered using a Butterworth filter. Features of acceleration and angular velocity were extracted by summarising the relevant research literature [24,25,26,27]. In this study, the time-domain and frequency-domain features (such as mean value, standard deviation, maximum value, total energy, mean square frequency and rectification mean value) of the six-axis data (acceleration and angular velocity) were extracted using a sliding window. Sliding windows should encompass at least one complete cycle of activity [28]. Using powers of 2 is advantageous for enhancing computational efficiency. Additionally, variations in activity cycles are considered among different subjects [29]. Based on the above-mentioned analysis, the sliding window was set to 2.56 s and the overlapping window was 50%. Thirty-two features were extracted from each axis, and 192 features were extracted from the six-axis data. The basic information (height, weight, age, gender, resting heart rate and average heart rate in each sliding window) was integrated, and each sample comprised 198 features; the corresponding label was associated with each sample, which was trained and verified using the algorithm model.

### 2.5. Model Introduction

#### 2.5.1. Classical Machine Learning Algorithms

In this study, because human basic information typically comprises discrete numerical data, the range of time-domain and frequency-domain features extracted from acceleration and angular velocity data may vary significantly. The K-nearest neighbour (KNN) algorithm does not require assumptions regarding data distribution and can handle various data types effectively. Additionally, it can adapt to different types of features. The decision tree (DT) algorithm is adept at handling data with mixed feature types and can automatically perform dimensionality reduction and eliminate redundant features, which is particularly useful for dealing with high-dimensional data. Given the favourable characteristics of KNN and DT algorithms, we chose these algorithms as representative examples of classic machine learning algorithms to construct models for cross-individual exercise intensity recognition. The KNN algorithm is capable of classification and prediction tasks [30] and is used to model data according to the similarity between various data features. The KNN algorithm projects various data features into the feature space and determines the training sample points in space by calculating the Euclidean distance, obtained using Equation (1):

(1) $$d\left(X,Y\right)=\sqrt{\overset{n}{\underset{k=1}{\sum }}{\left(x_{k}-y_{k}\right)}^{2}}$$

where $x_{k}$ and $y_{k}$ represent the *k* feature values of sample points *X* and *Y*, respectively.

**Figure 2:**
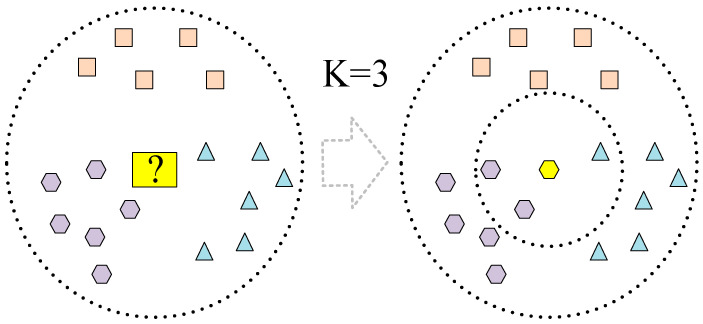
KNN algorithm structure diagram.

Finally, the sample feature points were projected from the test set into the training space, selecting the K training set samples closest to the test sample surroundings. The KNN algorithm determines which type of training sample appears most in the K training set samples, and the sample is classified as this type. The algorithm structure is shown in Figure 2. This study employed a grid search approach with five-fold cross-validation to determine the optimal K values for the seven designated body positions, as shown in Table 3.

DT is a tree-structure algorithm capable of classification and prediction tasks [31]. It is often employed as a foundational component for other intricate algorithms, such as random forest (RF) and gradient boosting decision tree (GBDT). DT is primarily constructed through a recursive approach until reaching a maximum depth or achieving a high level of purity in leaf nodes. Meanwhile, owing to the tendency of DTs to overfit, pruning is often employed to prevent overfitting. Therefore, when a test sample is input into a DT, classification can be performed by evaluating various features of the test sample. This study employed a grid search method with five-fold cross-validation to determine the optimal parameter combination for the seven designated body positions during modelling with the DT algorithm. The resulting parameter combination is presented in Table 3. For KNN, we performed a grid search over the hyperparameter n_neighbors, testing all integer values from 1 to 49. For decision trees (DTs), the search space included four hyperparameters: the splitting strategy (best, random), the impurity measure (gini, entropy), the maximum tree depth (1 to 9) and the minimum number of samples a leaf node can contain (1, 6, 11, …, 46).

#### 2.5.2. Ensemble Learning

**Figure 3:**
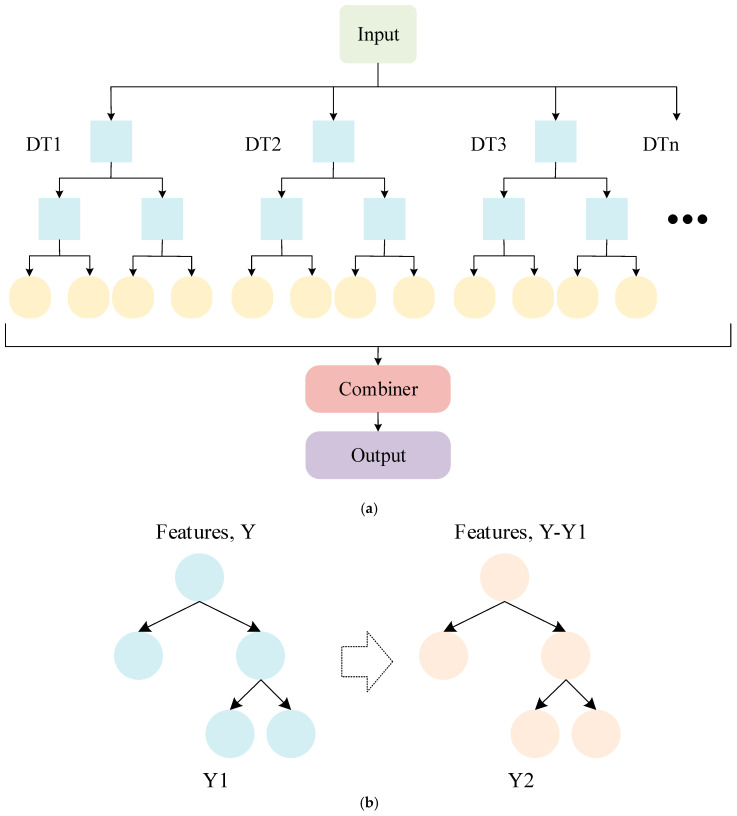
Network structure diagrams: (**a**) RF and (**b**) GBDT.

Ensemble learning combines multiple simple classifiers to form a model with stronger generalisation ability and better performance than when using individual classifiers alone, resulting in higher accuracy and robustness [32]. RF is composed of multiple DTs, and the output result of each time is determined by multiple DTs. In classification tasks, the final classification is determined by selecting the class that the majority of DTs choose. However, RF needs to build multiple DTs, so it requires more computing resources and time overhead [33]. GBDT is widely used in classification and prediction tasks, and it is composed of multiple DTs. During the model training process, continuous optimisation is conducted to improve the results of the previous tree, and the loss function is gradually used to fit the residuals, thereby improving the performance of the model [34]. The ability of GBDT to optimise DTs each time enables it to achieve higher accuracy and stronger generalisation ability. It can also assess the significance of features based on the construction of DTs. However, the computational cost and parameter adjustment can be substantial. The structures of the RF and GBDT algorithms are shown in Figure 3.

This study employed a grid search method with five-fold cross-validation to optimise the parameters of the RF and GBDT algorithms and determine the best parameters for each of the seven sensor placement positions. The optimal parameter combinations for these positions are detailed in Table 4 and Table 5. For GBDT, we explored a broad parameter space, including the learning rate (0.01, 0.05, 0.1, 0.15, 0.2, 0.25, 0.3), the n_estimators (50, 100, 150, 200, 250, 300), the max tree depth (3, 5, 6, 7, 8, 9, 10), the subsample (0.5, 0.6, 0.7, 0.8, 0.9, 1.0) and the max features for splitting (Auto, sqrt, log2). For RF, the grid search covered the n_estimators (50, 100, 150, 200), max depth (None, 2, 6, 10), minimum samples required to split a node (2, 5, 10), minimum samples per leaf (1, 2, 5) and the maximum features for splitting (sqrt, log2).

#### 2.5.3. Domain Adaptation

In traditional machine learning algorithms, it is often assumed that the distribution of one domain (source domain) is similar to that of another domain (target domain), and the source domain model is applied to the target domain. In this scenario, the source domain model exhibits good performance on the target domain. However, when there are disparities in the distribution between the source and target domains, it leads to rapid deterioration in model performance. Domain adaptation effectively addresses the issue of disparate distributions between the source and the target domains. It refers to the application of a model trained in the source domain to another related but different target domain. By learning the mapping relationship between the source domain and the target domain, the model gains better generalisation capabilities in the target domain and solves the problem of performance degradation caused by domain differences. The deep subdomain adaptation network (DSAN) is a deep learning algorithm designed to address disparities between domains. Unlike other domain adaptation methods that focus on global alignment, this algorithm adopts a more granular approach. This algorithm divides source domain data into multiple sub-domains by category and aligns the source and target domains with features in the sub-domains to reduce the deviation between the source and target domains. Lastly, the classifier is equipped with the ability to distinguish the features of the target domain and the source domain through adversarial training, thereby achieving domain adaptation. The core of the DSAN algorithm is to align the feature distributions of the same class in both the source and target domains through Local Maximum Mean Discrepancy (LMMD), as shown in Equation (2):

(2) $$d_{H}(p,q)≜E_{c}\|\;E_{p^{\left(c\right)}}[\phi (x^{s})]-E_{q^{\left(c\right)}}[\phi (x^{t})]{\|}_{H}^{2}$$

Here, $x^{s}$ and $x^{t}$ represent the feature representations of the source and target domains, while $p^{\left(c\right)}$ and $q^{\left(c\right)}$ represent the sub-domain distributions of class *c* in the source and target domains. This method measures the distribution difference between data, making the distributions of relevant sub-domains in the same class more similar.

Assuming classification can be achieved through the weights $w^{c}$ between different samples, the formula can be expressed as Equation (3):

(3) $${\hat{d}}_{H}\left(p,q\right)=\frac{1}{C}\overset{C}{\underset{c=1}{\sum }}{\left\|\underset{x_{i}^{s}\in D_{s}}{\sum }w_{i}^{sc}\emptyset \left(X_{i}^{s}\right)-\underset{x_{j}^{t}\in D_{t}}{\sum }w_{j}^{tc}\emptyset \left(X_{j}^{t}\right)\right\|}_{H}^{2}$$

where $w_{i}^{sc}$ and $w_{j}^{tc}$ represent the weights of different classes, and $\underset{x_{i}^{s}\in D_{s}}{\sum }w_{i}^{sc}\emptyset \left(X_{i}^{s}\right)$ and $\underset{x_{j}^{t}\in D_{t}}{\sum }w_{j}^{tc}\emptyset \left(X_{j}^{t}\right)$ represent the weighted sums on class c.

The weight w is calculated using Equation (4):

(4) $$w_{i}^{c}=\frac{y_{ic}}{\sum_{(x_{j},y_{j})\in D}y_{jc}}$$

where $y_{ic}$ represents the category label value of the i-th sample in the c-th sub-domain, and $\sum_{(x_{j},y_{j})\in D}y_{jc}$ represents the sum of the category label values of all samples in the dataset D in the c-th sub-domain.

To compute LMMD loss across multiple network feature layers, the source domain data is used with real labels, while the target domain data use soft labels predicted by the network. This can be expanded as shown in Equation (5):

(5) $${\hat{d}}_{l}(p,q)=\frac{1}{C}\sum_{c=1}^{C}\left[\begin{array}{l} \sum_{i=1}^{n_{s}}\sum_{j=1}^{n_{s}}w_{i}^{sc}w_{j}^{sc}k\left(z_{i}^{sl},z_{j}^{sl}\right)+\\ \sum_{i=1}^{n_{t}}\sum_{j=1}^{n_{t}}w_{i}^{tc}w_{j}^{tc}k\left(z_{i}^{tl},z_{j}^{tl}\right)-2\sum_{i=1}^{n_{s}}\sum_{j=1}^{n_{t}}w_{i}^{sc}w_{j}^{tc}k\left(z_{i}^{sl},z_{j}^{tl}\right) \end{array}\right]$$

where $z^{sl}$ and $z^{tl}$ represent the source and target domain features at layer l.

With reference to the structure of the DSAN network model [35], this study removed the feature extraction part of the convolutional neural network and determined the optimal hyperparameter combination through the grid search method. The hyperparameters of the network are listed in Table 6. For DSAN, we explored a broad hyperparameter space, including activation functions (ReLU, Leaky ReLU, ELU), optimisers (Adam, AdamW, RMSprop, SGD with momentum = 0.9), batch sizes (32, 64, 128, 256) and the number of neurons, among others. The Leaky ReLU is a variant of the ReLU activation function. The ReLU function outputs zero for all negative inputs, whereas the Leaky ReLU allows for a small negative slope in the negative region to prevent the neurons from becoming inactive. The Leaky ReLU improves the limitations of the traditional ReLU by introducing a small slope in the negative region, which is particularly beneficial in the backpropagation process to prevent the vanishing gradient problem. The formula for the Leaky ReLU is shown in Equation (6):

(6) f(x)=max(αx,x)

where 0 < α < 1.

The Leaky ReLU can alleviate the vanishing gradient problem and improve the generalisation ability of the model. By adjusting α, the gradient size for negative inputs can be controlled.

**Figure 4:**
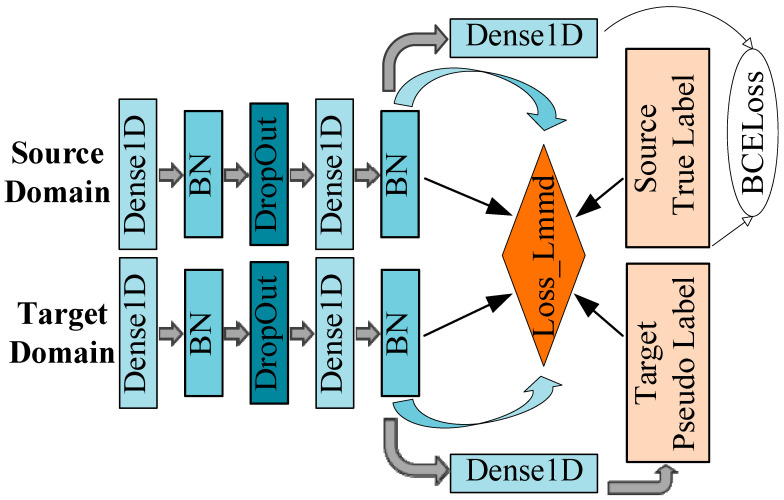
Network structure of DSAN.

The network structure diagram is shown in Figure 4.

#### 2.5.4. t-Distributed Stochastic Neighbour Embedding (t-SNE)

In this study, the sample set consists of high-dimensional data, and there is limited understanding of the data distribution, making it difficult to choose suitable algorithms. t-SNE can solve this problem. It enables the mapping of high-dimensional data to a lower-dimensional space through a non-linear process, maintaining similar relationships in high-dimensional spaces and thus achieving data dimensionality reduction [36]. First, t-SNE calculates the probability $p_{j|i}$ of point $x_{i}$ in high-dimensional space under the condition of point $x_{j}$, with the calculation in Equation (7) as follows:

(7) $$p_{j|i}=\frac{\exp\left(-\|x_{i}-x_{j}{\|}^{2}/2\sigma_{i}^{2}\right)}{\sum_{k\neq i}\exp\left(-\|x_{i}-x_{k}{\|}^{2}/2\sigma_{i}^{2}\right)}$$

$\sigma_{i}$ reflects the local scale parameter of point $x_{i}$, therefore achieving symmetry. The joint probability $p_{ij}$ is defined in Equation (8).

(8) $$p_{ij}=\frac{p_{j|i}+p_{i|j}}{2n}$$

where n represents the number of data points.

In low-dimensional space, the similarity probability $q_{ij}$ of the data points is calculated using Equation (9).

(9) $$q_{ij}=\frac{{\left(1+\|y_{i}-y_{j}{\|}^{2}\right)}^{-1}}{\sum_{k\neq l}{\left(1+\|y_{k}-y_{l}{\|}^{2}\right)}^{-1}}$$

Then, the optimal low-dimensional mapping is sought by minimising the Kullback–Leibler divergence between $p_{ij}$ and $q_{ij}$, as shown in Equation (10):

(10) $$KL(P\|Q)=\sum_{i\neq j}p_{ij}\log\frac{p_{ij}}{q_{ij}}$$

This achieves the mapping of high-dimensional data to low-dimensional space while preserving the local structure of the original data, thus fulfilling the purpose of dimensionality reduction and visualisation.

## 3. Results and Discussion

### 3.1. Data Analysis

**Figure 5:**
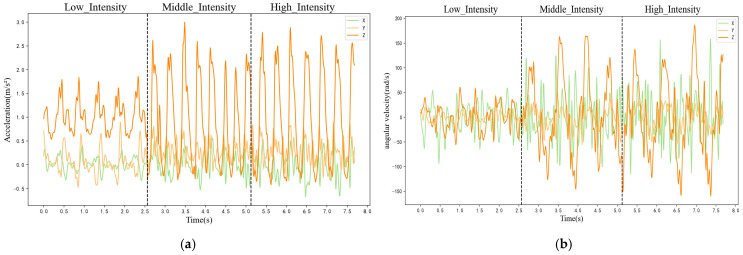
Line graph of waist acceleration and angular velocity in low-, middle- and high-intensity exercises: (**a**) triaxial acceleration; (**b**) triaxial angular velocity.

Taking the WitMotion sensor worn on the waist as an example, the data are preliminarily analysed and processed. The line chart of triaxial acceleration and triaxial angular velocity changing over time under low-, middle- and high-intensity exercises is drawn, and the time span of each exercise intensity stage is set to 2.56 s, as shown in Figure 5. It can be seen from the figure that the time-series curves of triaxial acceleration and triaxial angular velocity roughly change periodically, and the amplitude difference between low intensity and middle and high intensity is large, while the amplitude difference between middle intensity and high intensity is small.

**Figure 6:**
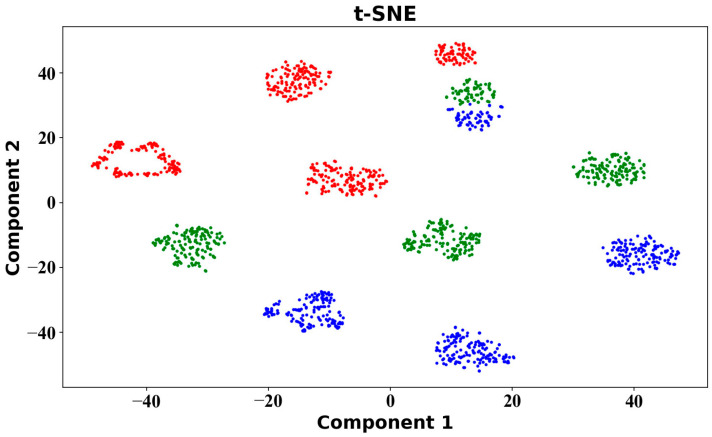
Visualised motion data graph for t-SNE: red, green and blue indicate low-, middle- and high-intensity exercises, respectively.

To further explore the distribution of data, data from four participants were randomly selected from the waist dataset and visualised by the t-SNE dimensionality reduction algorithm. The visual result of dimensionality reduction is shown in Figure 6, showing noticeable variations in data distribution among individuals, which can be attributed to individual differences, resulting in each person’s data not conforming to a uniform distribution. Therefore, further analysis of data is needed in the stage of cross-individual exercise intensity recognition. In this study, we will embark on data modelling from the perspectives of both classic machine learning algorithms and domain adaptation methods.

### 3.2. Comparison of Exercise Intensity Recognition of Different Parts Based on Classical Machine Learning Algorithms

This study took running as an example to divide the exercise intensity into three levels, collected the motion sensor data of different body parts during running and constructed the exercise intensity recognition model using two classical machine learning algorithms. The specific parameters of the algorithm model are elaborated in detail in Section 2.5.1. This study collected exercise data from a total of 24 participants. Models were constructed using a six-fold cross-validation method based on individual partitioning, and the final accuracy of the model was determined by averaging the accuracies of the six models. The proportions of gender and age in both groups were randomised to avoid any bias from these factors, providing a more accurate assessment of the model’s true performance.

This study constructed two classic machine learning algorithm models for cross-individual exercise intensity recognition using the training sets. The training set and test set were sequentially input into the models, and model accuracies on the test set and training set are presented in Table 7. The overall performance of the KNN algorithm model and the DT algorithm model was relatively close, and the recognition rate of the left limb was mostly higher than that of the right limb. The KNN algorithm performed better in the lower extremity than in the upper extremity. This observation was further supported by six-fold cross-validation, which consistently revealed higher accuracy rates in lower limb regions than their upper limb counterparts, as detailed in Supplementary Table S1. Among the 14 models established based on different body positions and algorithms, the KNN model exhibited the best performance for the left_calf position, achieving a recognition rate of 82.97%. Overall, the accuracy on the training set was higher than that on the testing set, with a considerable difference, indicating that there is a certain difference in the data distribution between the training set and the test set. The aforementioned results demonstrated the feasibility of employing classical machine learning algorithms for cross-individual exercise intensity recognition. However, these algorithms were slightly insufficient in dealing with cross-individual exercise intensity.

### 3.3. Comparison of Exercise Intensity Recognition of Different Parts Based on Ensemble Learning

**Figure 7:**
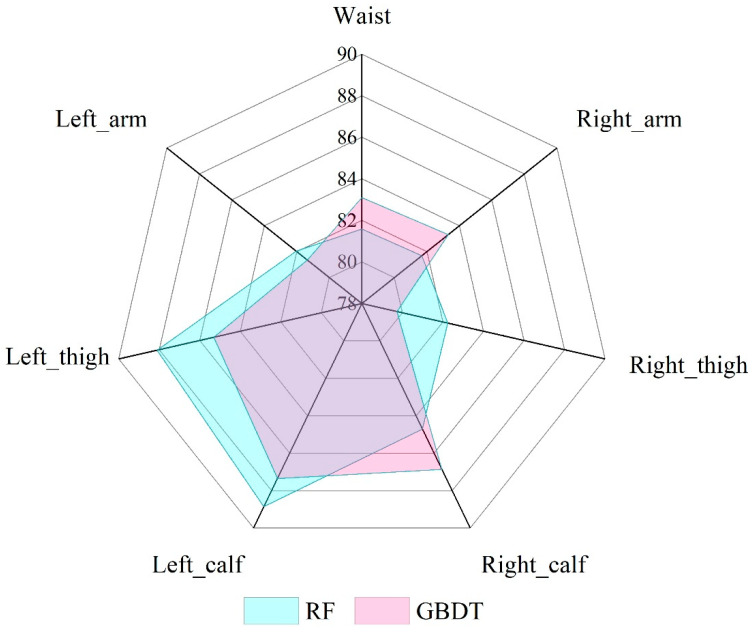
Comparison of accuracies of the RF and GBDT algorithms on the test set.

This study employed two ensemble learning algorithm models, namely RF and GBDT, for cross-individual exercise intensity recognition. The parameters of these two ensemble learning algorithm models have been elaborated in Section 2.5.2. In this study, the training sets and test sets were put into the model using the same six-fold cross-validation method, and the recognition rates of the RF and GBDT algorithm models were finally obtained, as shown in Figure 7. In both the RF and GBDT algorithm models, the accuracy on the training set reached 100%, demonstrating their excellent classification capability for cross-individual exercise intensity data. When comparing the recognition performance on the test set, the average accuracy of the RF and GBDT algorithms was 84.18% and 83.86%, respectively. The model based on the left limb data exhibited better recognition performance than the model based on the right limb data. The RF algorithm model exhibited better performance than the GBDT algorithm model. Their average recognition accuracies for the three exercise intensities were 84.18% and 83.86%, respectively. The recognition rate based on the left_calf position was the best in both models, with accuracy rates of 88.86% and 87.35%, respectively. As shown by the test data, although ensemble learning algorithm models outperformed classical machine learning algorithms for the recognition of cross-individual exercise intensity, a disparity exists between the accuracy of the models on the test set and the training set, necessitating further investigation and research.

### 3.4. Comparison of Exercise Intensity Recognition at Different Parts Based on Domain Adaptation

**Figure 8:**
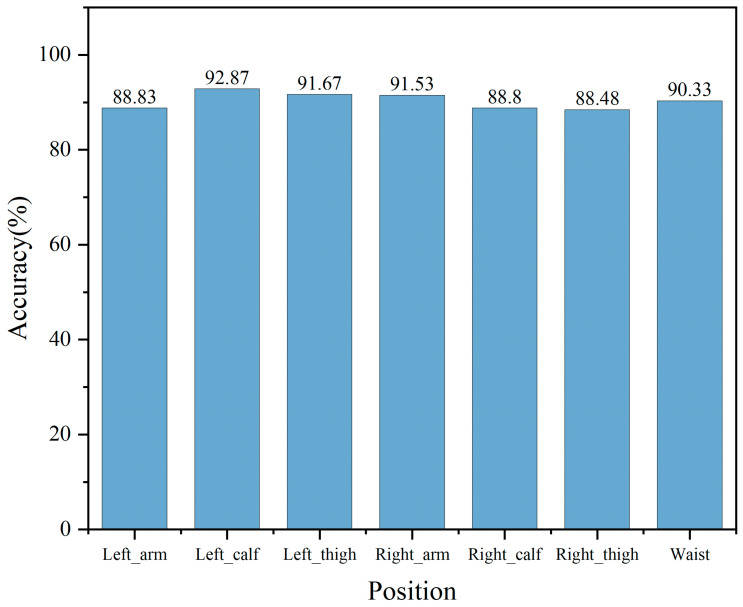
Accuracies of the DSAN algorithm on the testing set and training set.

In this study, the DSAN algorithm model was used to determine cross-individual exercise intensity, and its hyperparameters have been described in Section 2.5.3. The data of four participants were taken as the target domain dataset, and the data of 20 participants were taken as the source domain dataset; the features of the two datasets were aligned according to the category. Finally, the target domain dataset was put into the algorithm model, and the accuracies for detecting exercise intensities at the seven parts of the target domain were obtained, as shown in Figure 8. Among the seven parts, the lowest recognition rate was observed in the right thigh, with a recognition rate of 88.48%. The highest recognition rate was found in the left calf, boasting a recognition rate of 92.87%, highlighting excellent recognition performance. The overall performance is mainly better in the left part sensor model, which may be because the left part is a non-habitual part, so there will be fewer redundant actions, and its stability is high, greatly reducing interference. The DSAN algorithm model, unlike the traditional machine learning and ensemble learning methods, achieved an accuracy of 90.36%. This indicates that the DSAN algorithm model can overcome the challenges posed by individual variations.

**Figure 9:**
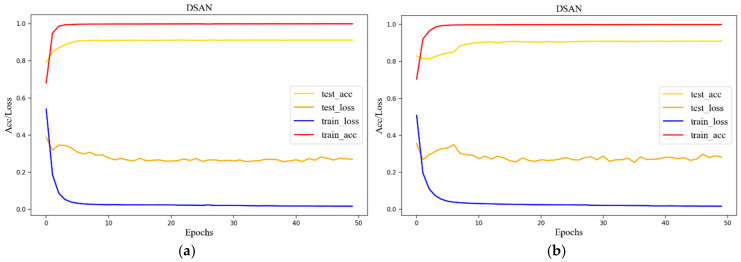
Accuracies and loss curves of the test set and training set: (**a**) left calf of the participant; (**b**) right calf of the participant.

Taking the left calf and right calf as examples, the variations in loss values and accuracies of the DSAN algorithm on the test and training sets are shown in Figure 9. The results show that the loss and accuracy when using the training set quickly reached a balance. Meanwhile, the loss and accuracy when using the test set were slightly lower than the rate at which the training set reached equilibrium. The test and training set convergence was rapid, yet the loss value when using the test set could not approach zero. This may be because the training and testing sets could not follow a uniform distribution, even though they were aligned as much as possible according to categories. Some individual differences may have a significant impact, or the feature dimensions may not be sufficient for complete differentiation, resulting in discrepancies between the results of the testing and training sets.

**Figure 10:**
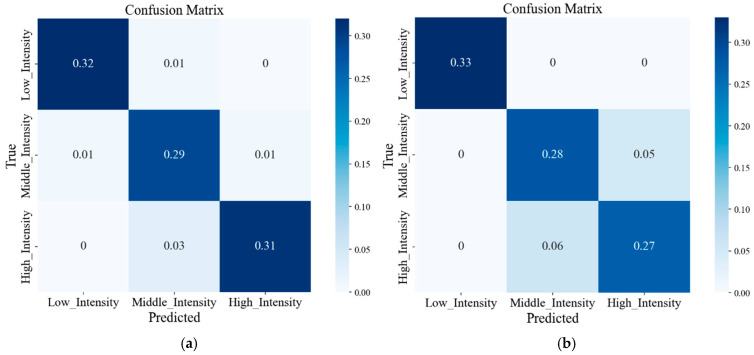
Confusion matrix of exercise intensity: (**a**) left_calf and (**b**) right_thigh.

To further analyse the performance of the model, we drew the confusion matrix of the two parts of the body with the highest and lowest accuracies, as shown in Figure 10. Then, precision, recall and F1-score were calculated through the confusion matrix, as shown in Table 8. Overall, in the two confusion matrices, the identification error rate for the low intensity was lower, whereas higher error rates were observed for the middle and high intensities. In the low-intensity classification, the right thigh exhibited outstanding performance. However, the middle- and high-intensity classifications were prone to errors, as evidenced by their precision, recall and F1-score. In the identification of the left calf, although the low-intensity classification did not exhibit outstanding performance compared to that of the right thigh, the overall results were satisfactory, with most indicators surpassing 0.9, reflecting the excellent performance of this part.

### 3.5. Discussion

In this study, a line chart of the six-axis inertial sensor data of the subjects was drawn, and the line chart showed roughly periodic changes. The difference between low-intensity and middle- and high-intensity exercises was large, which could be intuitively obtained from the amplitude, while the difference between middle- and high-intensity exercises was small. This study used the t-SNE visualisation method to draw the motion feature maps of participants and found that the data between different participants did not completely follow a uniform distribution. The classical machine learning algorithm, ensemble learning algorithm and DSAN algorithm model were used to verify the cross-individual exercise intensity recognition, and the accuracy of the sensor on the left body part was found to be generally higher than that on the right body part. The average accuracy on the test set for the KNN and DT algorithms was 78.02% and 77.26%, respectively, while for RF and GBDT, it was 84.18% and 83.86%. This indicated that the two classical machine learning algorithms performed poorly in cross-individual exercise intensity recognition. The highest recognition rate on the test set was only 82.97%, which differed significantly from the recognition rate on the training set. The performance of the ensemble learning algorithm model was better than that of the classical machine learning algorithm model, and the highest recognition rate was 88.86%. The DSAN algorithm model exhibited the best performance, with the highest recognition rates across all body parts. Among all body parts, the highest recognition rate was observed in the left calf, reaching an accuracy of 92.87%. According to various indicators, the DSAN algorithm model demonstrated strong stability, highlighting the outstanding advantages of this model in addressing cross-individual scenarios.

## 4. Conclusions

In daily life, exercise intensity is a crucial metric that people typically pay attention to during physical activities, and the accurate monitoring of low-, middle- and high-intensity exercises is of great significance to people’s health. Therefore, this study proposed to use easily collected multi-dimensional human body data to identify low-, middle- and high-intensity exercises. t-SNE visualisation results showed that the exercise data of most people do not conform to a unified distribution, indicating that individual differences have a certain impact on the data distribution. The use of classical machine learning algorithm models for cross-individual exercise intensity recognition showed poor performance. Meanwhile, the performance of ensemble learning algorithm models was slightly better than that of classical machine learning algorithm models. However, the accuracy exhibited a considerable gap compared to that when using the training set, indicating a difficulty for both ensemble learning and machine learning algorithms in handling cross-individual recognition. This is because their recognition premise is that the data follow a uniform distribution. The DSAN algorithm was capable of effectively handling data that did not conform to a uniform distribution. This algorithm represented a more fine-grained method, outperforming several other algorithm models, and the highest recognition rate reached 92.87%. Thus, DSAN excellently addressed the issue arising from individual variations. All algorithmic models consistently indicated that the recognition performance in the left body parts was superior to that in the right body parts. Simultaneously, misclassifications often occurred in middle- and high-intensity exercises, probably due to the subtle distinctions between middle- and high-intensity exercises. This study establishes a theoretical foundation and exhibits practical significance for the identification of exercise intensity through the combination of multi-dimensional sensors and domain adaptation methods.

This study has several limitations. First, the grading of exercise intensity is not sufficiently detailed. Second, the age range distribution of participants for data collection is relatively narrow. Third, the establishment of cross-individual exercise intensity recognition methods is not comprehensive enough. Therefore, in future studies, more detailed classifications of exercise intensity levels should be established, and data from more people should be collected for exercise intensity monitoring. Moreover, using additional feature extraction methods, more rich features can be extracted, and the cross-individual deep algorithm model can be optimised to achieve improved recognition effects.

## Figures and Tables

**Table 1:** Basic information of participants.

Variable	Men (*n* = 12)	Women (*n* = 12)
Age (yr)	21.92 ± 1.89	22.92 ± 2.18
Weight (kg)	74.69 ± 6.09	59.87 ± 9.20
Height (cm)	181.04 ± 7.62	165.08 ± 5.78
Rest rate (bpm)	66.42 ± 6.74	70.92 ± 11.54

**Table 2:** Exercise intensity data standards in running.

Exercise Intensity	Standard (%VO_2max_)	Time	Intensity Level Label
Low intensity	0–45%	4 min	0
Middle intensity	46–63%	4 min	1
High intensity	64–100%	4 min	2

**Table 3:** Optimal parameter combinations for the KNN and DT algorithms.

	KNN	DT			
	K	Splitter	Criterion	Max_Depth	Min_Samples_Leaf
Left_arm	8	best	entropy	9	1
Right_arm	5	best	entropy	9	1
Waist	5	best	entropy	9	1
Left_thigh	3	best	entropy	9	1
Right_thigh	5	best	entropy	9	1
Left_calf	3	best	entropy	9	1
Right_calf	3	best	entropy	9	1

**Table 4:** Optimal parameter extraction results for the RF algorithm.

	Max_Depth	Max_Features	Z_Leaf	Z_Split	n_Estimators
Left_arm	None	sqrt	1	2	200
Right_arm	None	sqrt	1	2	200
Waist	None	sqrt	1	2	100
Left_thigh	None	sqrt	1	2	200
Right_thigh	None	sqrt	1	2	100
Left_calf	None	sqrt	1	5	150
Right_calf	None	sqrt	1	2	150

Note: Z stands for Min_samples; the term “sqrt” represents that the number of features considered at each split point is set to the square root of the total number of features.

**Table 5:** Optimal parameter extraction results for the GBDT algorithm.

	Subsample	n_Estimators	Max_Features	Max_Depth	Learning_Rate
Left_arm	1	150	Auto	8	0.2
Right_arm	1	150	Auto	8	0.2
Waist	1	300	Auto	5	0.05
Left_thigh	0.6	100	Auto	8	0.25
Right_thigh	1	100	Auto	8	0.25
Left_calf	0.7	150	log2	10	0.25
Right_calf	0.6	100	Auto	8	0.25

**Table 6:** DSAN network hyperparameters.

Name	Parameter	Description
Learning Rate	0.0001	Controls the magnitude of weight updates
Epoch	50	Maximum number of iterations for training
Batch Size	32	The number of samples in an iteration
Optimiser	AdamW	Updating network weights
Loss Function	Binary CrossEntropy Loss	Measures the gap between predicted and actual values
Activation Function	Leaky_ReLU	Introduces non-linearity
Learning Rate Decay	Learning Rate Scheduler	Improves performance

**Table 7:** Accuracies of the KNN and DT algorithm test and training sets.

	KNN		DT	
	Train	Test	Train	Test
Left_arm	98.90%	72.70%	99.27%	79.42%
Right_arm	99.15%	73.33%	99.24%	78.20%
Waist	99.67%	74.67%	99.59%	77.13%
Left_thigh	99.97%	82.01%	99.83%	77.51%
Right_thigh	99.92%	80.87%	99.55%	71.48%
Left_calf	99.97%	82.97%	99.92%	80.31%
Right_calf	99.98%	79.58%	99.60%	76.80%

**Table 8:** Model evaluation indicators for left_calf and right_thigh.

		Precision	Recall	F1-Score
Left_calf	Low	0.97	0.97	0.97
	Middle	0.88	0.94	0.91
	High	0.97	0.91	0.94
Right_thigh	Low	1	1	1
	Middle	0.82	0.85	0.83
	High	0.84	0.82	0.83

## Data Availability

The data presented in this study are available on request from the corresponding author.

## Supplementary Materials

The following supporting information can be downloaded at: https://www.mdpi.com/article/10.3390/s25113437/s1, Table S1: Test set accuracy in six-fold cross-validation.

## Abbreviations

RPE	rating of perceived exertion
TT	talk test
%HRR	percentage of heart rate reserve
ACSM	American College of Sports Medicine
KNN	K-nearest neighbour
DT	decision tree
RF	random forest
GBDT	gradient boosting decision tree
DSAN	deep subdomain adaptation network
t-SNE	t-distributed neighbour embedding
