# Supplementary material for: A Feasibility Study of Domain Adaptation for Exercise Intensity Recognition Based on Wearable Sensors
Source: Sensors (Basel). 2025 May 30;25(11):3437. doi: 10.3390/s25113437 (PMC12158358; doi:10.3390/s25113437)
Supplement: Supplementary file 1 [file sensors-25-03437-s001.zip › sensors-3567638-supplementary.pdf]

Table S1 Test set accuracy in six-fold cross-validation

	Left_arm	Right_arm	Left_thigh	Right_thigh	Left_calf	Right_calf
fold 1	0.654776	0.760465	0.716889	0.933445	0.834056	0.768774
fold 2	0.726138	0.802089	0.817669	0.797447	0.902844	0.862955
fold 3	0.774212	0.747227	0.767482	0.774482	0.785482	0.74879
fold 4	0.717265	0.641279	0.78576	0.693809	0.785559	0.820527
fold 5	0.742643	0.754971	0.880197	0.852713	0.779643	0.775306
fold 6	0.746682	0.693595	0.952462	0.800111	0.890463	0.798487
